# Buprenorphine Markedly Elevates a Panel of Surrogate Markers in a Murine Model of Sepsis

**DOI:** 10.1097/SHK.0000000000001361

**Published:** 2019-10-15

**Authors:** Weiqiang Chen, Max Brenner, Monowar Aziz, Sangeeta S. Chavan, Clifford S. Deutschman, Betty Diamond, Valentin A. Pavlov, Barbara Sherry, Ping Wang, Kevin J. Tracey, Haichao Wang

**Affiliations:** ∗ The Feinstein Institute for Medical Research, Northwell Health, Manhasset, New York; † Donald and Barbara Zucker School of Medicine at Hofstra/Northwell, Hempstead, New York

**Keywords:** Analgesics (buprenorphine), inflammation, sepsis, sepsis surrogate markers

## Abstract

Sepsis can be simulated in animals by perforating the cecum via a surgical procedure termed “cecal ligation and puncture” (CLP), which induces similar inflammatory responses as observed during the clinical course of human sepsis. In addition to anesthetic agents, many Institutional Animal Care and Use Committees often recommend the use of additional analgesic agents (such as opioid) to further augment the initial anesthetic effects. However, emerging evidence suggest that a commonly recommended opioid, buprenorphine, dramatically elevated circulating interleukin (IL)-6 levels, and reduced animal survival in male C57BL/6 mice, but not in female mice possibly due to the complex interference of estrous cycles, fueling an ongoing debate regarding the possible impact of analgesic administration on the sepsis-induced systemic inflammation. As per the recommendation of a local government agency, we performed a pilot study and confirmed that repetitive administration of buprenorphine indeed markedly elevated circulating levels of four sepsis surrogate markers (e.g., IL-6, KC, monocyte chemoattractant protein-1, and granulocyte-colony stimulating factor) in 20% to 60% of septic animals. This complication may adversely jeopardize our ability to use the CLP model to reliably simulate human sepsis, and to understand the complex mechanism underlying the pathogenesis of lethal sepsis. Thus, for experimental sepsis studies set to survey systemic inflammation and animal lethality at relatively later stages (e.g., at 24 h post CLP and beyond), we strongly recommend not to repetitively administer buprenorphine to eliminate its potential complication to animal sepsis models.

## INTRODUCTION

Sepsis is defined as life-threatening organ dysfunction caused by a dysregulated host response to infection (1). It can be simulated in animals by perforating the cecum via a surgical procedure termed “cecal ligation and puncture” (CLP) (2), which causes bacteria spillage to the peritoneal cavity, and mimics perforated appendicitis or diverticulitis in humans. The severity of experimental sepsis, as reflected by the systemic accumulation of a panel of surrogate markers (e.g., interleukin [IL-6], KC, monocyte chemoattractant protein-1 [MCP-1], and granulocyte-colony stimulating factor [G-CSF]) and parallel animal mortality, can be artificially controlled by varying the size of the needle used to puncture the cecum (3). In animals, CLP induces similar inflammatory responses as observed during the clinical course of human sepsis (2), and is thus regarded as the most clinically relevant animal model for human sepsis syndrome (2, 3).

In addition to injectable (e.g., ketamine and xylazine) and inhaled (e.g., isoflurane) anesthetic agents, many Institutional Animal Care and Use Committees (IACUC) often recommend the additional use of analgesic agents (such as opioid or nonsteroidal anti-inflammatory drug [NSAID]) to further augment the initial anesthetic effects. However, extensive literature has suggested a substantial impact of NSAID, such as flubiprofen (4) or ibuprofen (5), on the sepsis-induced inflammation and lethality. Similarly, Cotroneo et al. (6) recently reported that a commonly recommended opioid, buprenorphine, when given subcutaneously at 0.1 mg/kg immediately pre- and 12 h post CLP, dramatically elevated circulating IL-6 levels (by 13-fold), and reduced animal survival (from 70% to 10%) in male C57BL/6 mice. In female mice, however, this analgesic agent did not obviously affect the sepsis-induced systemic inflammation and lethality (6, 7), possibly due to the complex interference of estrous cycles (8). Thus, it has become a topic of ongoing debate whether analgesic administration adversely influences CLP sepsis-induced systemic inflammation in mice. As per the recommendation of the New York State Department of Health, we performed a pilot study to evaluate the effect of analgesics on sepsis-induced systemic inflammation by comprehensively surveying 62 circulating cytokines and chemokines at 28 h post CLP.

## MATERIALS AND METHODS

### Animal model of sepsis

This pilot study was conducted in accordance with the policies of the NIH Guide for the Care and Use of Laboratory Animals, and approved by the IACUC of the Feinstein Institute for Medical Research (in an amendment to the animal protocol #2017-027, approved on November 28, 2018). Male BALB/c mice (8–10 wks, 20 g–25 g, Taconic Biosciences, Inc., Germantown, NY) were anesthetized intramuscularly with ketamine (75 mg/kg, Henry Schein Animal Health, Dublin, Ohio) and xylazine (10 mg/kg, Akorn Inc, Lake Forest, Ill) before subjecting the surgical area to hair shaving and sterilization with providone iodine. A 15-mm midline incision was made to expose the cecum, and a 4-0 PROLENE Polypropylene Suture ligature was placed 5.0 mm from the cecal tip. The ligated cecal stump was then punctured once with a 22-gauge needle without direct extrusion of stool, and immediately returned back to its normal intra-abdominal position. After closing the abdomen wound with staples (wound clips), all animals were immediately given a small amount of bupivacaine (0.25%, 50 μL–100 μL, Hospira Inc, Lake Forest, Ill) locally around the incision site, and followed by a subcutaneous injection of imipenem/cilastatin (0.5 mg/mouse) (Primaxin, Merck & Co Inc, West Point, Pa) 30 min post CLP. Afterward, all animals were supplemented with supportive care by resuscitating with normal sterile saline solution (20 mL/kg), and provided with gel diet on the floor of the cages for septic animals’ easy access.

### Buprenorphine regimen

To confirm the impact of buprenorphine on sepsis-induced inflammation, animals were randomly divided into four groups, and given only saline (“Bup 0”), or buprenorphine (Bup, 0.1 mg/kg, Reckitt Benckiser Healthcare (UK) Ltd, Hull, UK) immediately before CLP (“Pre-CLP”), or followed with repeated dosing every 12 h (i.e., 12 and 24 h post CLP) for a total of one to three injections (“Bup 1, Bup 2, Bup3”). Control mice received three injections of equal volume of saline at equivalent time points.

Bup 0: CLP + Saline n = 5
Bup 1: CLP + Bup (once: Pre-CLP) n = 5
Bup 2: CLP + Bup (twice: Pre-CLP, +12 h post CLP) n = 5
Bup 3: CLP + Bup (thrice: Pre-CLP, + 12 h, + 24 h post CLP) n = 5

We used this dosing regimen as per the recommendation of our institutional veterinarian, reasoning that at a lower dose (e.g., 0.05 mg/kg, every 12 h) buprenorphine might not provide sufficient analgesia to septic animals.

### Cytokine antibody array

At 28 h post CLP, animals in all four groups (“CLP + Saline,” “CLP + Bup 1,” “CLP + Bup 2,” and “CLP + Bup 3”) were euthanized by CO_2_ asphyxiation, and blood was harvested by terminal cardiac puncture. Murine Cytokine Antibody Arrays (Cat. No. M0308003, RayBiotech Inc, Norcross, Ga) were used to determine the serum levels of 62 cytokines and chemokines, as previously described (9, 10). Briefly, the membranes were sequentially incubated with equal volume of serum samples, primary biotin-conjugated antibodies, and horseradish peroxidase-conjugated streptavidin. After exposing to X-ray film, the relative signal intensity for each cytokine or chemokine (in duplicate) was determined using the Scion Image software, and expressed as the percentage of positive controls on respective membranes.

## RESULTS AND DISCUSSION

**Fig. 1:**
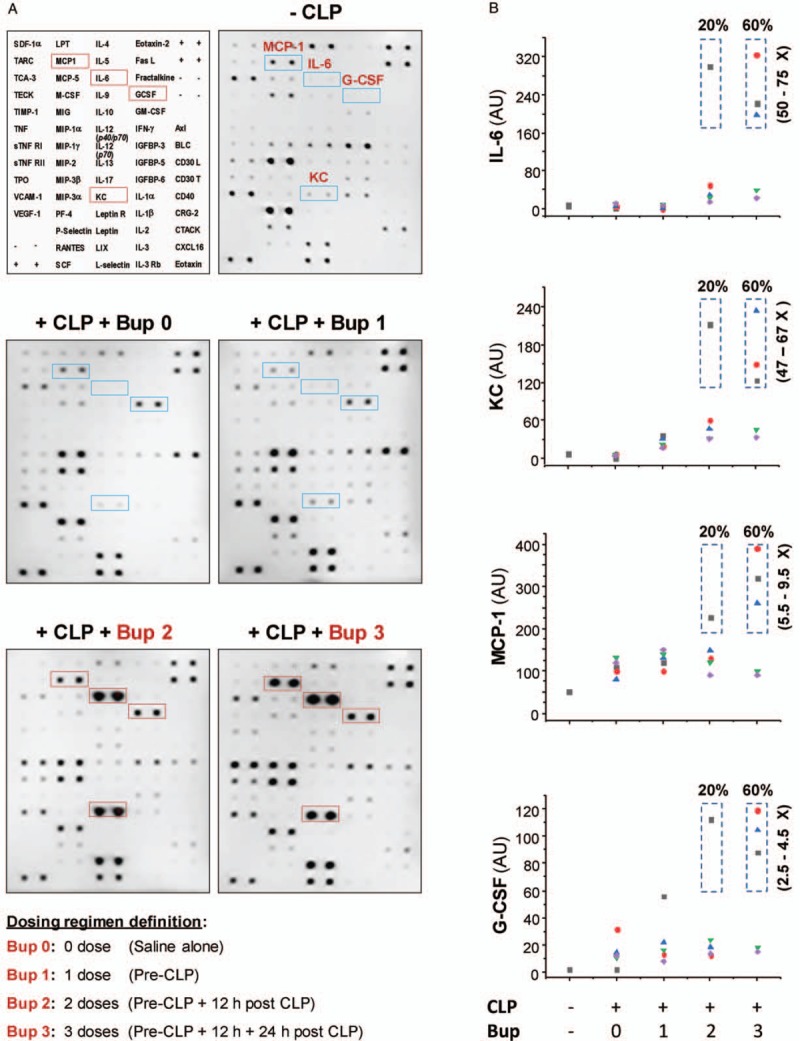
Repetitive administration of buprenorphine markedly elevates circulating levels of four surrogate markers of lethal sepsis.

A single dose of buprenorphine given immediately pre-CLP surgery did not obviously affect serum levels of various cytokines or chemokines at a relatively late time point—28 h post CLP (Fig. 1,A and B). However, repeated administration of buprenorphine (twice: pre-CLP and 12 h post CLP) markedly elevated serum levels of IL-6, KC, MCP-1, and G-CSF in one out of five (20%) animals (Fig. 1B). Furthermore, repetitive administrations of buprenorphine (thrice: pre-CLP, 12 and 24 h post CLP) dramatically elevated serum levels of these four cytokines/chemokines in three out of five (60%) animals (Fig. 1B).

A, Representative Cytokine Antibody Arrays showing serum levels of 62 cytokines and chemokines. B, Quantitative analysis of relative serum levels of IL-6, KC, MCP-1, and G-CSF at 28 h post CLP. Note that the serum levels of several surrogate markers for lethal sepsis were markedly elevated in 1/5 (20%) and 3/5 (60%) of mice treated with two and three doses of buprenorphine at indicated time points. Marked by the dashed boxes were the levels of four sepsis surrogate markers that were markedly elevated by repetitive administration of buprenorphine (“Bup”). Numbers in the parentheses indicated the fold (“X”) increase over the “+ CLP + Saline” group.

Notably, IL-6, KC, MCP-1, and G-CSF have been regarded as sepsis surrogate markers, the levels of which could reliably predict the lethal outcome of experimental (11, 12) and clinical sepsis (13). Using the same model of CLP sepsis, we previously demonstrated that circulating levels of IL-6, KC, MCP-1, and G-CSF were dramatically higher in animals that were approaching a moribund state (i.e., unresponsive to physical stimuli, incapable of maintaining upright position, and with agonal breathing) than those remaining in the non-moribund state (i.e., responsive to physical stimuli, capable of maintaining upright position, and with normal breathing) at + 52 h post CLP (14). Thus, our findings suggest that repetitive administration of buprenorphine at a commonly recommended dose (e.g., 0.1 mg/kg) markedly elevates circulating levels of four surrogate markers of lethal sepsis in 20% to 60% of the animals. Our findings were consistent with a recent finding that a single dose of buprenorphine did not affect sepsis-induced systemic accumulation of MCP-1 and IL-6 at 24 h post CLP (15), but repetitive buprenorphine administration markedly elevated blood IL-6 level in male B57BL/6 mice at a similar time point (6).

For many centuries, chronic pain has forced patients to seek opioid pain therapies, but its overuse has recently spiraled into an opioid epidemic that currently costs billions of dollars and thousands of lives annually in the United States alone. Ever since its initial inception as an active ingredient of poppy seeds in 1805, morphine has been widely used as a potent opioid (narcotic) analgesic for relieving chronic pain. However, its clinical use is hindered by several side effects, including the induction of tolerance, severe withdrawal symptoms, as well as high risks of addiction and abuse. Thus, there have been continuous efforts to make morphine analogs with minimized side effects, culminating in the recent development of buprenorphine as an effective narcotic replacement therapy for treating opioid addiction (16). Unlike morphine, buprenorphine acts as a partial agonist that binds with high affinity to, but only partially activates, the μ (mu)-opioid receptors (MOR) of the pain-inhibitory pathway. However, it also behaves as an antagonist for the δ (delta) and κ (kappa) opioid receptors (DOR and KOR) (17). The lack of DOR-agonist activity might account for the long-sought-after mechanism underlying the diminishment of buprenorphine tolerance after prolonged use. It is thus theoretically possible that a single dose of buprenorphine acts as analgesic, but repetitive administration might lead to the occupancy of multiple opioid receptors, thereby simultaneously activating both anti-analgesic KOR and analgesia- and allodynia-inducing DOR *in vivo*. However, it is currently unknown whether the possible divergent receptor occupancy after single versus multiple doses of buprenorphine administration contributes in any way to the observed difference in buprenorphine-mediated cytokine/chemokine enhancement following different dosing regimens.

Our current study also has several other limitations. First, we do not know whether a single dose of buprenorphine given immediately pre-CLP could also affect the sepsis-induced early inflammatory response. This was because we harvested blood samples at a relatively later time point (28 h post CLP), reasoning that some septic animals soon succumb to death thereafter. It might be important to measure circulating cytokines/chemokines at multiple time points (6–36 h post CLP) to gain a comprehensive view of buprenorphine-mediated dynamic effects on the sepsis-induced inflammation. Second, the mechanism by which repetitive buprenorphine administration markedly elevated sepsis surrogate markers was not investigated. In light of previous findings that morphine worsens the outcome of experimental sepsis by impairing gut barrier integrity to enhance bacterial spillage to the blood and other organs (18), it might be important to investigate whether buprenorphine elevates systemic inflammation similarly by disrupting intestinal barrier function to trigger bacterial translocation in future studies. Moreover, it might also be necessary to determine whether repetitive buprenorphine administration similarly affects other clinical endpoints including tissue bacterial load, organ function, and survival in experimental and clinical sepsis.

Realizing the challenge we are facing to balance the needs of sepsis research against the welfare of the animals used in the experimentation (19), our institutional IACUC members and sepsis investigators diligently exchange information, and carefully weigh the possible confounding effects of analgesics on the CLP sepsis model against the moral obligation to perform humane research for eventually improving human health. Reasoning that CLP surgery produces a systemic inflammation model that causes gradual neuropathic pain during the sepsis progression, we continue to alleviate initial surgical pain with local anesthetic (e.g., bupivacaine), but now propose to omit additional buprenorphine use to alleviate subsequent pain associated with the disease progression of sepsis. This is because repetitive use of buprenorphine in the CLP model paradoxically elevated both sepsis surrogate markers (Fig. 1) and animal lethality through complicated mechanisms (6), which not only leads to unnecessary use of more animals per experimental group, and but also jeopardizes our ability to use CLP to reliably simulate human sepsis, or to understand its complex pathogenic mechanisms. Thus, for experimental sepsis studies set to survey systemic inflammation and animal lethality at relatively later stages (e.g., at 24 h post CLP and beyond), we strongly recommend not to repetitively administer buprenorphine to eliminate its potential complication to animal sepsis models. Of course, for animal experiments that need to monitor early inflammatory responses, additional pilot studies should be performed to determine whether a single dose of buprenorphine given immediately pre-CLP could also affect sepsis-induced inflammation at earlier stages.

Finally, it is not yet known whether it is time to warn septic patients not to use buprenorphine (BELBUCA) as an alternative pain therapy, because buprenorphine might similarly propagate excessive systemic inflammation in clinical settings. This is relevant, because a recent retrospective study revealed a prevalent use of opioids in septic patients (20). Moreover, septic patients who received opioid therapy appeared to exhibit a significantly increased risk of 28-day mortality than those without opioid therapy (10.4% vs. 2.4%) (20), although patients receiving opioids might be relatively sicker at the beginning, and potentially biased this retrospective study toward a relatively worse 28-day outcome. Nevertheless, buprenorphine (BELBUCA) should not be prescribed to patients with various diseases such as severe asthma, intestinal and bowel blockage, and perhaps also severe sepsis.
